# Comparative Evaluation of Cartridge-Based Abbott ID NOW Test With Probe-Based Real-Time Reverse Transcription Polymerase Chain Reaction Assay for Detection of SARS-CoV-2

**DOI:** 10.7759/cureus.22470

**Published:** 2022-02-21

**Authors:** Dipti Pattnaik, Nirmala Poddar, Basanti K Pathi, Kumudini Panigrahi, Smaranita Sabat, Ankita Roy, A. Raj K Patro, Amrut Mohapatra, Shubhransu Patro, Ashok K Praharaj

**Affiliations:** 1 Microbiology, Kalinga Institute of Medical Sciences, Bhubaneswar, IND; 2 Community Medicine, Institute of Medical Sciences and SUM Hospital (IMS and SUM Hospital), Bhubaneswar, IND; 3 Molecular Biology, Kalinga Institute of Medical Sciences, Bhubaneswar, IND; 4 Pulmonary Medicine, Kalinga Institute of Medical Sciences, Bhubaneswar, IND; 5 Department of General Medicine, Kalinga Institute of Medical Sciences, Bhubaneswar, IND

**Keywords:** sensitivity, lamp assay, id now, rt-pcr, sars-cov-2, covid-19, pandemic

## Abstract

Background: The gold standard test for detection of severe acute respiratory syndrome coronavirus 2 (SARS-CoV-2) recommended by WHO is real-time reverse transcription polymerase chain reaction (RT-PCR), which has a turnaround time of five to six hours. Abbott ID NOW (Abbott Diagnostics Scarborough, Inc., Scarborough, ME, USA), the cartridge-based loop-mediated isothermal amplification (LAMP) assay, was approved by FDA for Emergency Use Authorization as rapid point of care testing. The present study was planned to evaluate the performance of the cartridge-based Abbott ID NOW test by comparing it to the currently used standard probe-based real-time RT-PCR method for detection of SARS-CoV-2.

Methodology: A cross-sectional study was conducted in a tertiary care hospital in the eastern part of India after getting institutional ethics committee (IEC) approval. Two hundred fifty-nine cases of various age groups of both sexes who were advised for testing for SARS-CoV-2 were included in the study. Nasopharyngeal swabs were collected according to protocol advisory by the Indian Council of Medical Research (ICMR), India. Dry swabs were sent for Abbott ID NOW testing and swabs in viral transport medium were sent for probe-based RT-PCR assay using the CoviPath kit (Thermo Fisher Scientific, Bangalore, India). The data were collected and statistical analysis was performed using Statistical Package for Social Sciences (SPSS) (IBM Corp., Armonk, NY, USA). Sensitivity, specificity, positive and negative predictive values for ID NOW were calculated taking RT-PCR as the gold standard.

Results: Out of 259 patients enrolled in the study, 49% were symptomatic for coronavirus disease 2019 (COVID-19). The prevalence rate of SARS-CoV-2 was 20.84% among the study population. Sensitivity and specificity, positive and negative predictive values of ID NOW test in comparison to RT-PCR assay was found to be 87%, 98%, 92.1% and 96.8% respectively. ID NOW detected seven out of 54 (12.9%) cases as false negative who were found to be positive with RT-PCR, with mean Ct value of the target genes >34.

Conclusions: In this study the overall sensitivity for ID NOW assay was found to be lower, but specificity, positive and negative predictive values were found to be higher. It had the highest correlation to RT-PCR among symptomatic patients and at higher viral loads. Due to the ease of use and shortest result time for detecting COVID-19, ID NOW test could be used as a point-of-care test. But for all tests, the results should be interpreted according to the clinical and epidemiological context.

## Introduction

In December 2019, a novel coronavirus, severe acute respiratory syndrome coronavirus 2 (SARS-CoV-2), causing severe respiratory symptom (coronavirus disease 2019 [COVID-19]) emerged as a global pandemic from Wuhan, China [1]. Globally as of 31 Jan 2022, there have been 373,229,380 confirmed cases of COVID-19 including 5,658,702 deaths [2]. SARS-CoV-2 infected individuals may be asymptomatic or may have range of symptoms, varying from mild upper respiratory tract illness or gastrointestinal disorder to severe respiratory distress with multi-system failure and death [3]. Accurate and rapid laboratory diagnosis of the cases became necessary entity during the rapidly developing crisis for identifying the infected individuals, contact tracing, epidemiological characterization and public health decision making.

In response to the rapidly evolving COVID-19 pandemic, the FDA granted emergency use authorization allowing the use of various molecular assay for in vitro diagnosis of COVID-19. However the performance characteristics of the authorized assays are not well studied and analyzed [4].

SARS-CoV-2 infection is confirmed by detection of SARS-CoV-2 RNA in patient samples by using nucleic acid amplification tests (NAAT). Among different NAATs, detection of SARS-CoV-2 RNA by the real-time reverse transcriptase polymerase chain reaction (RT-PCR) is recommended as the most sensitive method [5,6].

SARS-CoV-2 gene targets used to detect SARS-CoV-2 by RT PCR include nucleocapsid (N), envelope (E), spike (S), RNA-dependent RNA polymerase (RdRp) and open reading frame1ab (ORF1ab) genes [6,7]. WHO recommends real-time RT-PCR as the gold standard test for SARS-CoV-2 identification and routine confirmation. The RT-PCR test takes four to six hours for detection of SARS-CoV-2. However, due to the given situation of COVID-19 pandemic, the time needed to get the test result, to reduce test load of RT-PCR in different setups and rapid need of a reliable report, point-of-care testing has an advantage in terms of ease of use and over technically demanding real-time RT-PCR [5,8].

Loop-mediated isothermal amplification (LAMP) has the advantages of point-of-care testing in rapid amplification at a single temperature and can be used in rapid detection of SARS-CoV-2, suitable for use in the clinic with the least trained personnel [8]. It is claimed that limit of detection for SARS-CoV-2 in Abbott ID NOW (Abbott Diagnostics Scarborough, Inc., Scarborough, ME, USA) is 125 genome equivalents/ml of sample. It has less turnaround time (15-30 minutes) and is more patient friendly [8,9].

The number of commercially available point-of-care tests for SARS-CoV-2 is increasing. Commonly available Abbot ID NOW assay uses isothermal nucleic acid amplification of the RNA-dependent RNA polymerase (RdRp) viral gene target [7-9].

This study aimed to evaluate the performance of cartridge-based Abbott ID NOW test by comparing to the currently used probe-based real-time RT-PCR (gold standard) method for detection of SARS-CoV-2.

## Materials and methods

A cross-sectional study was conducted in the Department of Microbiology of a tertiary care hospital in the eastern part of India after obtaining ethical approval from the Institutional Ethics Committee (Ref. No.: KIIT/ KIMS/ IEC/ 728/2021). All patients irrespective of age and gender, who attended to out-patient department (OPD), fever clinic and emergency department presented to the Kalinga Institute of Medical Sciences (KIMS), Bhubaneswar, for whom a physician had advised the test for SARS-CoV-2, during the period of July 2021 to September 2021 were included in the study. Relevant demographic and clinical data were collected about them in case record format.

Two sets of nasopharyngeal swabs (NPS) were collected from each patient using flocked NPS and one of the NPS was transported to the laboratory in universal viral transport media (VTM) within one to two hours of collection in cold chain for open RT-PCR testing. The second sample was sent to the laboratory at room temperature as a dry swab in the same sleeve as that in which the swabs were packaged for cartridge-based ID NOW testing. 

The dry NPS were tested within two hours of collection or kept refrigerated at 4 to 8°C for up to 24 hours before testing as per package insert instructions. The dry swab was subjected to sample lysis in lysis buffer. Automated isothermal amplification of the nucleotides and binding of target was done in a single cartridge of Abbott ID NOW. Qualitative detection of SARS-CoV-2 viral nucleic acid was done and results displayed on the screen were noted down.

The swab sent in VTM was subjected for processing and RNA extraction in Thermo Fisher RNA extraction system (Thermo Fisher Scientific, Bangalore, India) using KingFisher Flex Model. CoviPath COVID-19 RT-PCR Kit (Thermo Fisher Scientific) was used to detect SARS-CoV-2 by real-time RT-PCR protocols following the manufacturer’s instructions on a QuantStudio 5 real-time PCR-System (Applied Biosystems, Waltham, MA, USA). The assay includes two target regions of SARS-CoV-2 gene sequences: N gene and ORF1ab. Further, one target RNase P was included as an internal control to monitor the sample source. The results were interpreted using QuantStudio 5-DX software (Thermo Fisher Scientific) according to the manufacturer’s instructions. A specimen was considered SARS-CoV-2 positive when two SARS-CoV-2 gene targets were positive with cycle threshold (Ct) values of ≤37.

Demographic and clinical data were expressed in mean and percentage. Sensitivity, specificity, positive and negative predictive values for ID NOW were calculated taking RT-PCR as the gold standard. Statistical analysis was performed using Statistical Package for Social Sciences (SPSS) (IBM Corp., Armonk, NY, USA).

## Results

A total of 259 patients were enrolled in the study, with mean age of 43 years, out of which 153 (59%) were male and 106 (41%) were female. Prevalence rate of SARS-CoV-2 was 54/259 (20.84%) among the study population and 49/128 (38.28%) patients presented with COVID-19 symptoms (Table 1).

**Table 1:** Distribution of demographic data among patients advised for COVID-19 testing COVID-19: coronavirus disease 2019; SARS-CoV-2: severe acute respiratory syndrome coronavirus 2; RT-PCR: reverse transcription polymerase chain reaction

Demographic data	Value
Sex	Male- 153 (59 %)
	Female-106 (41%)
Mean age	43 years
Symptomatic for COVID-19 symptoms	128/ 259 (49.4%)
SARS CoV-2 RT PCR Positive	54 /259 (20.84%)
Total (N)	259

**Figure 1:**
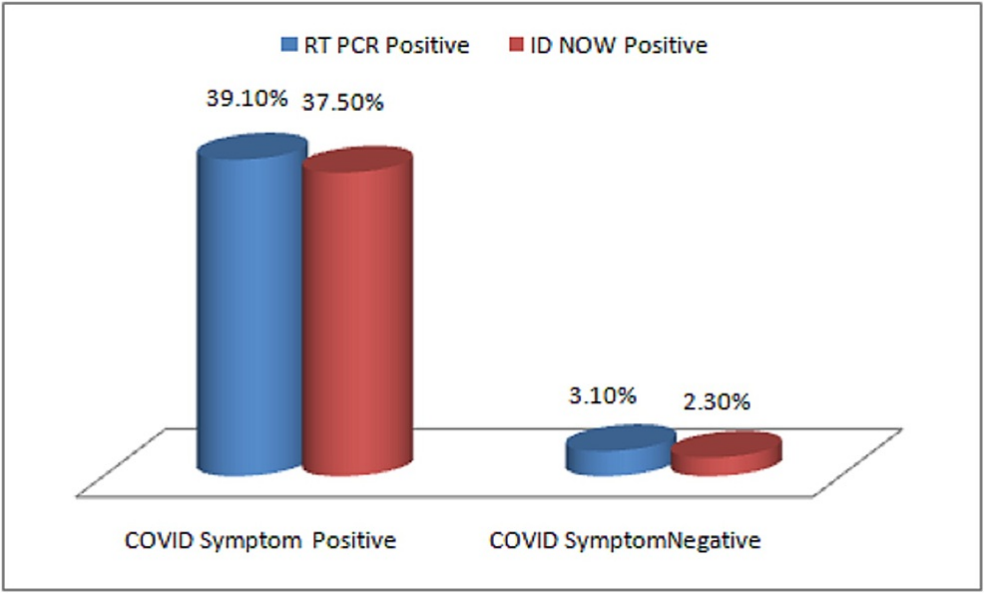
Variation of RT-PCR and ID NOW test results with COVID-19 symptoms in detection of SARS-CoV-2 COVID-19: coronavirus disease 2019; SARS-CoV-2: severe acute respiratory syndrome coronavirus 2; RT-PCR: reverse transcription polymerase chain reaction

On analysis of different test methods for SARS-CoV-2 detection, it was found that 54/259 (20.84%) positive cases detected by RT-PCR method, 47/54 (87.03 %) cases were detected as positive by both ID NOW and RT-PCR assay. ID NOW detected seven of 54 (12.9%) cases as false negatives and four cases as false positives in comparison to the RT-PCR assay (Figure 1, Table 2).

**Table 2:** Comparison of ID NOW test with RT-PCR assay on NPS for detection of SARS-CoV-2 COVID-19: coronavirus disease 2019; SARS-CoV-2: severe acute respiratory syndrome coronavirus 2; RT-PCR: reverse transcription polymerase chain reaction; NPS: nasopharyngeal swab

	RT-PCR Positive	RT-PCR Negative	Total
ID NOW Positive	47	4	51
ID NOW Negative	7	201	208
Total	54	205	259

Sensitivity and specificity of the ID NOW test in comparison to the gold standard RT-PCR test were found to be 87% and 98% respectively. Positive predictive value (PPV) and negative predictive value (NPV) for ID NOW were found to be 92.1% and 96.8% respectively.

On clinical evaluation of both test results, it was found that among symptomatic patients RT-PCR assay detected 50/128 (39.1%) cases and ID NOW method detected 48/128 (37.5%) and both the tests were found to be statistically significant in detection of COVID-19 with p value < 0.05.

ID NOW method could not detect SARS-CoV-2 among seven patients showing false-negative results, who were found to be positive with RT-PCR, with Ct value of the target genes >34. Also in four cases, ID NOW showed false-positive results which were found to be negative by RT PCR, even after repeating the test for confirmation. All four cases were asymptomatic patients.

Results of ID NOW compared with the mean Ct values of target genes obtained in RT-PCR are shown in Table 3. At low Ct value ID NOW results were in 100% concordance with RT-PCR results. 

**Table 3:** Comparison of ID NOW results with mean Ct value of RT-PCR test Ct: cycle threshold; RT-PCR: reverse transcription polymerase chain reaction

Ct Value of Target Genes	Mean Ct Value ORF1 ab gene		Mean Ct Value N gene	Number of RT PCR tests positive	Number of ID NOW positives
11-20	16.6	15.38		17	17
21-30	24.30	25.78		26	26
>31	34.36	34.52		11	4

Turnaround times for the tests were recorded in this study, which were found to be 15-30 minutes for ID NOW test in comparison to the five to six hours turnaround time of the RT-PCR assay. 

## Discussion

The advent of the COVID-19 pandemic created a unique situation worldwide, where the FDA granted emergency use authorization allowing the use of several molecular diagnostic assays for detection of SARS-CoV-2. However, the performance characteristics of the authorized assays are not well studied or analyzed. Although the sensitivity and specificity of assays for the detection of SARS-CoV-2 have not been systematically evaluated, currently RT-PCR method is regarded as the gold standard to which other testing methodologies can be compared. But the cartridge-based Abbott ID NOW technology is helpful to provide rapid and accurate results in our institution, especially for our emergency department as point-of-care testing.

The present study was planned to evaluate the performance of cartridge-based Abbott ID NOW assay by comparing it with the gold standard probe-based RT-PCR assay for detection of SARS-CoV-2 among patients who were advised for COVID-19 testing by the clinicians during the study period.

A total of 259 patients were enrolled in the study, the majority (59%) were male patients and 49.4% were symptomatic for COVID-19. The age and sex distribution of COVID-19 cases found in the present study are similar to the findings of other studies [10,11]. Gupta et al. [11] in their study mentioned that age group 21 to 50 years with male sex were commonly affected by COVID-19.

In this study, in both nucleic acid-based detection assays, ID NOW and RT-PCR detected 87.03% positive cases whereas 12.9% cases were found to be false negative and four (3.1%) cases were found to be false positive by ID NOW test. The present study found sensitivity and specificity of the ID NOW test in comparison to the gold standard RT-PCR test as 87% and 98% respectively among our population. Positive predictive value and negative predictive value for ID NOW were found to be 92.1% and 96.8% respectively. Other studies also mentioned similar findings [12].

Farfour et al. [13] in their study found that overall positive predictive agreement of 73.9% with ID NOW, when compared against the cobas Roche assay (Roche, Basel, Switzerland). Tu et al. [14] in his study compared paired nasopharyngeal samples by ID NOW COVID-19 assay and Abbott RT-PCR method; the positive predictive agreement of ID NOW to RT-PCR was 74.73% and the negative predictive agreement was 99.41%. ID NOW assay in this study showed a very high specificity of 98%, which is in agreement with the manufacturer’s claimed specificity of over 97%.

On clinical evaluation of both test results, it was found that among symptomatic patients RT-PCR assay detected 39.1% cases and ID NOW method detected 37.5% and both the tests found to be statistically significant in detection of COVID-19 with p value < 0.05. He et al. [15] in their study mentioned that the highest viral load in throat swabs was found at the time of symptom onset, which shows that at high viral load both the NAAT assays could produce significant results. Also the limit of detection of RT-PCR assays is lower than that of the isothermal ID NOW assay, so the infected persons will remain positive for significantly longer times even after becoming symptomatic, after the time of peak viral load [14,12].

While comparing Ct values of RT-PCR with ID NOW results, it was observed that at low Ct values ID NOW results were in 100% concordance with RT-PCR results. Performance of the ID NOW decreased for samples with high Ct values. Other studies also suggested that the sensitivity of ID NOW was reduced as the Ct values exceeded 34.5 [12-14]. Patients having lower levels of SARS-CoV-2 in the nasopharynx might harbor even lower concentrations in the nasal cavity, leading to a lower level of detection by the ID NOW.

The cause behind differences in detection of SARS-CoV-2 by the ID NOW and CoviPath RT-PCR assay used in our study may be due to different principles used for both the NAAT technologies. Basu et al. [12] in their study mentioned that isothermal nucleic acid amplification test is a promising tool for detection of SARS-CoV-2, but having lower sensitivity than RT-PCR for SARS-CoV-2 RNA detection.

Our study has limitations. The heterogeneity of samples including outpatients and emergency from a single center, relatively small sample size, variability in sampling technology. Further, studies with larger sample size from different centers are warranted for evaluation of the performance of ID NOW test.

The present study was done to evaluate the performance of the cartridge-based Abbott ID NOW test by comparing it with the gold standard RT-PCR test. In this study, it was observed that the turnaround time from sample collection to dispatch of report for ID NOW test was 15-30 minutes which was much less in comparison to the five- to six-hour turnaround time of RT-PCR assay. So ID NOW test is more suitable for giving point-of-care testing in emergency departments.

## Conclusions

Our study found that sensitivity of the ID NOW test is lower but specificity is high. Due to the ease of use and shortest result time for detecting COVID-19, ID NOW can be used as a rapid point of care test for diagnosis of COVID-19 with samples from symptomatic patients at high viral load, patients coming to emergency department, but there is the possibility that ID NOW may miss infections in the asymptomatic infected population with low viral load. However negative results should be correlated with clinical symptoms and radiological evidence of COVID-19.

## References

[1] Hu B, Guo H, Zhou P, Shi ZL (2021). Characteristics of SARS-CoV-2 and COVID-19. Nat Rev Microbiol.

[2] (2022). WHO-Coronavirus Dashboard. https://covid19.who.int.

[3] Wiersinga WJ, Rhodes A, Cheng AC, Peacock SJ, Prescott HC (2020). Pathophysiology, transmission, diagnosis, and treatment of coronavirus disease 2019 (COVID-19): a review. JAMA.

[4] Mitchell SL, St George K, Rhoads DD, Butler-Wu SM, Dharmarha V, McNult P, Miller MB (2020). Understanding, verifying, and implementing emergency use authorization molecular diagnostics for the detection of SARS-CoV-2 RNA. J Clin Microbiol.

[5] Kevadiya BD, Machhi J, Herskovitz J (2021). Diagnostics for SARS-CoV-2 infections. Nat Mater.

[6] Islam KU, Iqbal J (2020). An update on molecular diagnostics for COVID-19. Front Cell Infect Microbiol.

[7] Mathuria JP, Yadav R, Rajkumar Rajkumar (2020). Laboratory diagnosis of SARS-CoV-2 - a review of current methods. J Infect Public Health.

[8] Shen M, Zhou Y, Ye J, Abdullah Al-Maskri AA, Kang Y, Zeng S, Cai S (2020). Recent advances and perspectives of nucleic acid detection for coronavirus. J Pharm Anal.

[9] Ramachandran A, Noble J, Deucher A, Miller S, Tang PW, Wang RC (2021). Performance of Abbott ID-Now rapid nucleic amplification test for laboratory identification of COVID-19 in asymptomatic emergency department patients. J Am Coll Emerg Physicians Open.

[10] Population Pyramid.net (2020 (2020). Population Pyramids of the World from 1950 to 2100 - India. https://www.populationpyramid.net/india/2020.

[11] Gupta MK, Bhardwaj P, Goel AD, Saurabh S, Misra S (2021). Trends of epidemiological and demographic indicators of COVID-19 in India. J Infect Dev Ctries.

[12] Basu A, Zinger T, Inglima K (2020). Performance of Abbott ID Now COVID-19 rapid nucleic acid amplification test using nasopharyngeal swabs transported in viral transport media and dry nasal swabs in a New York City academic institution. J Clin Microbiol.

[13] Farfour E, Asso-Bonnet M, Vasse M (2021). The ID NOW COVID-19, a high-speed high-performance assay. Eur J Clin Microbiol Infect Dis.

[14] Tu YP, Iqbal J, O'Leary T (2021). Sensitivity of ID NOW and RT-PCR for detection of SARS-CoV-2 in an ambulatory population. Elife.

[15] He X, Lau EH, Wu P (2020). Temporal dynamics in viral shedding and transmissibility of COVID-19. Nat Med.

